# Interleukin 10 (IL-10): an immunosuppressive factor and independent predictor in patients with metastatic renal cell carcinoma

**DOI:** 10.1038/sj.bjc.6690189

**Published:** 1999-03

**Authors:** F Wittke, R Hoffmann, J Buer, I Dallmann, K Oevermann, S Sel, T Wandert, A Ganser, J Atzpodien

**Affiliations:** Department of Hematology and Oncology, Medizinische Hochschule Hannover, Hannover, Germany; Department of Experimental Immunology, Institut Necker, Paris, France; Department of Cell Biology and Immunobiology, National Centre for Biotechnology (GBF), Mascheroder Weg 1, Brannschweig, Germany

**Keywords:** predictors, renal cell carcinoma, IL-10

## Abstract

Interleukin 10 (IL-10) is an immunosuppressive factor and has been detected in tumour cell cultures of renal cell carcinoma and of malignant melanoma. IL-10 has been described as a cytokine of the Th2 response; it is able to suppress antigen-presenting cells (APCs) and may lead to down-regulation of HLA class I and II molecules on dendritic cells and to anergy of T-lymphocytes. We evaluated pretreatment serum levels of soluble IL-10 and various clinical parameters to determine their prognostic value in 80 advanced renal cell carcinoma patients seen at our institution between May 1990 and April 1996. For statistical evaluation we used both univariate and multivariate Cox proportional hazards models. An elevated pretreatment serum level of IL-10 was a statistically independent predictor of unfavourable outcome (*P* < 0.0028), in addition to the well-known clinical and biochemical risk factors. These data support risk stratification for future therapeutic trials and identify a predictor which needs to be validated in prospective studies and may potentially influence decision making in palliative management of patients with metastatic renal cell carcinoma. These data also suggest a potential role of IL-10 in the development of advanced renal cell carcinoma and in the future design of therapeutic strategies. © 1999 Cancer Research Campaign


*British Journal of Cancer* (1999) **79**(7/8), 1182–1184
© 1999 Cancer Research Campaign
Article no. bjoc.1998.0189

### Interleukin 10 (IL-10): an immunosuppressive factor and independent predictor in patients with metastatic renal cell carcinoma

**F Wittke[1], R Hoffmann, J Buer[2,*], I Dallmann[1], K Oevermann, S Sel, T Wandert[1], A Ganser[1] and J Atzpodien[1]**

[1]Department of Hematology and Oncology, Medizinische Hochschule Hannover, Germany; [2]Department of Experimental Immunology, Institut Necker, Paris, France; *Department of Cell Biology and Immunobiology, National Centre for Biotechnology (GBF) , Mascheroder Weg 1, Brannschweig, Germany

**Summary** Interleukin 10 (IL-10) is an immunosuppressive factor and has been detected in tumour cell cultures of renal cell carcinoma and of malignant melanoma. IL-10 has been described as a cytokine of the Th2 response; it is able to suppress antigen-presenting cells (APCs) and may lead to down-regulation of HLA class I and II molecules on dendritic cells and to anergy of T-lymphocytes. We evaluated pretreatment serum levels of soluble IL-10 and various clinical parameters to determine their prognostic value in 80 advanced renal cell carcinoma patients seen at our institution between May 1990 and April 1996. For statistical evaluation we used both univariate and multivariate Cox proportional hazards models. An elevated pretreatment serum level of IL-10 was a statistically independent predictor of unfavourable outcome ($P < 0.0028$), in addition to the well-known clinical and biochemical risk factors. These data support risk stratification for future therapeutic trials and identify a predictor which needs to be validated in prospective studies and may potentially influence decision making in palliative management of patients with metastatic renal cell carcinoma. These data also suggest a potential role of IL-10 in the development of advanced renal cell carcinoma and in the future design of therapeutic strategies.

**Keywords: predictors, renal cell carcinoma, IL-10**

Interleukin 10 (IL-10), originally defined as cytokine synthesis inhibitory factor produced by type 2 helper T-lymphocytes (Fiorentino et al, 1989), has subsequently been described as a potentially immunosuppressive cytokine. By interfering with the co-stimulatory function of antigen-presenting cells (APCs), e.g. regulation of class II MHC expression of monocytes (deWaal Malefyt et al, 1991), and co-stimulatory molecule expression of macrophages (Ding et al, 1993), IL-10 reduces antigen-specific T-cell proliferation. Moreover, a direct effect of IL-10 on IL-2 production by activated T-cells has been observed (deWaal Malefyt et al, 1993). It has been demonstrated that IL-10 blocks the development of predendritic cells into mature dendritic cells (DCs) in vitro (Buelens et al, 1997).

This IL-10-mediated immunosuppression represents an important escape mechanism of tumours and needs further clinical investigation.

Therefore, using a highly sensitive enzyme-linked immunosorbent assay (ELISA) recognizing both human and viral IL-10, we measured the serum concentrations of IL-10 in 80 consecutive patients with a fully documented clinicopathological history of metastatic renal cell carcinoma seen in our institution since May 1990. We assessed the potential correlation between serum IL-10 levels and the outcome of these patients.

### METHODS

### Patients and collections of samples

This study was approved by the institutional review board of the Medizinische Hochschule Hannover; written informed consent was obtained from all patients prior to entry into the study. At this time, we obtained samples of peripheral blood from 80 consecutive patients with metastatic renal cell carcinoma, seen at our institution at the Medizinische Hochschule Hannover, Germany since May 1990. Sera were frozen at –80°C until analysis.

Patient characteristics are summarized in Table 1; all patients had a Karnovsky performance status ≥70%, and presented with histologically confirmed metastatic renal cell carcinoma and with clinically progressive disease as demonstrated by standard radiographic procedures. Patients received systemic immunotherapy with subcutaneous IL-2 and interferon-alpha (Atzpodien et al, 1995); treatment was continued until disease progression occurred. Survival was measured from start of systemic therapy.

### ELISA for IL-10

Pretreatment levels of soluble IL-10 were determined using an ELISA assay (IL-10, Medgenix, Ratingen, Germany). All analyses were performed in duplicate, strictly according to the procedures recommended by the manufacturers, and samples were analysed at a dilution resulting in measured concentrations within the range of the standard curves. Normal donor sera showed no measurable IL-10 serum levels in this assay. Sensitivity was at 0.208 pg ml$^{-1}$ with a range of 0.78–50 pg ml$^{-1}$. Cross-reactivity was specified with less than 1%.

### Statistical analysis

The statistical end point in our analysis was overall survival from time of entry into the study. We calculated univariate hazard ratios with 95% confidence intervals, using the Cox proportional hazards model. The simultaneous prognostic effect of various factors was determined in a multivariate analysis using a Cox

*Received 17 February 1998*
*Revised 12 June 1998*
*Accepted 22 June 1998*

*Correspondence to:* J Atzpodien, Department of Hematology and Oncology, Medizinische Hochschule Hannover, 30625 Hannover, Germany

**Table 1** Patient characteristics

| Variable | *n* | IL-10 ≤ 1 pg ml⁻¹ *n*[a] | IL-10 > 1 pg ml⁻¹ *n* |
|---|---|---|---|
| Patients | 80 | 59 | 21 |
| Sex | | | |
| Male | 60 | 44 | 16 |
| Female | 20 | 15 | 5 |
| Age (years) | | | |
| Mean | 57 | 56 | 57 |
| Range | 32–74 | 37–72 | 32–74 |
| Metastases | | | |
| Lung | 59 | 43 | 16 |
| Bone | 23 | 17 | 6 |
| Liver | 20 | 13 | 7 |
| Brain | 11 | 8 | 3 |
| Tumour sites | | | |
| 1 | 28 | 24 | 4 |
| > 1 | 52 | 35 | 17 |

[a]Serum IL-10 levels were assayed prior to systemic therapy.

**Table 2** Impact of pretreatment serum IL-10 levels and clinical factors on patient survival in 80 consecutive patients with advanced renal cell carcinoma by univariate analysis

| Variable | Categories compared[a] | *P*-value |
|---|---|---|
| Clinical factors | | |
| Sex | Female vs. male | 0.89 |
| Age (years) | < 60 vs. ≥ 60 | 0.155 |
| Time between diagnosis and progression (months) | > 12 vs. ≤ 12 | 0.16 |
| Haemoglobin (g dl⁻¹) | ≥ 10 vs. < 10 | 0.0000 |
| ESR (mm h⁻¹) | < 50 vs. ≥ 50 | 0.0004 |
| LDH (U l⁻¹) | < 240 vs. ≥ 240 | 0.016 |
| Lung metastases | Present vs. absent | 0.86 |
| Bone metastases | Absent vs. present | 0.18 |
| Liver metastases | Absent vs. present | 0.92 |
| Brain metastases | Absent vs. present | 0.12 |
| Local relapse | Absent vs. present | 0.35 |
| IL-10 (pg ml⁻¹) | ≤ 1 vs. > 1 | 0.0028 |

[a]For each variable, the prognostic significance of the first category listed was assessed by comparing that category with the reference category (the second category listed).

proportional hazards model (forward and reverse selection of variables). The probability of overall survival was plotted over time according to the method of Kaplan and Meier (Kaplan et al, 1958). Differences in overall survival between groups were tested as dichotomized prognostic variables. For IL-10, erythrocyte sedimentation rate (ESR) and haemoglobin, Kaplan–Meier estimates were performed defining the best cut-off value for discrimination between poor and good overall survival. For lactate dehydrogenase (LDH), the institutional upper normal limits were chosen as the cut-off (240 U l⁻¹).

### RESULTS

### Univariate analysis of pretreatment variables and survival

We analysed the ability of various clinical factors and of serum levels of soluble IL-10 to predict clinical outcome. The mean period of follow-up for the surviving patients was 32+ months (range, 2–80+ months). The median survival of all 80 patients entering the study was 21+ months.

We calculated univariate *P*-values with the Kaplan–Meier model (Table 2). In this analysis, IL-10, LDH, haemoglobin and ESR had significant prognostic impact on overall survival. Elevated serum levels of IL-10 (> 1 pg ml⁻¹) were detected in 21 patients and were strongly associated with an unfavourable outcome. Median survival in these patients was 11+ months as opposed to patients with low IL-10 levels (≤ 1 pg ml⁻¹, $n$ = 59) who had a median survival of 27+ months ($P < 0.0028$).

All other variables investigated (sex, age, time between diagnosis and progression, lung metastases, bone metastases, liver metastases, brain metastases and local relapse) were not statistically significant for the prediction of patient survival.

### Multivariate analysis of risk factors

To identify the most powerful independent predictors, we established a multivariate Cox proportional hazards model containing those factors with significant prognostic value upon univariate analysis. All four univariate risk factors were found to be

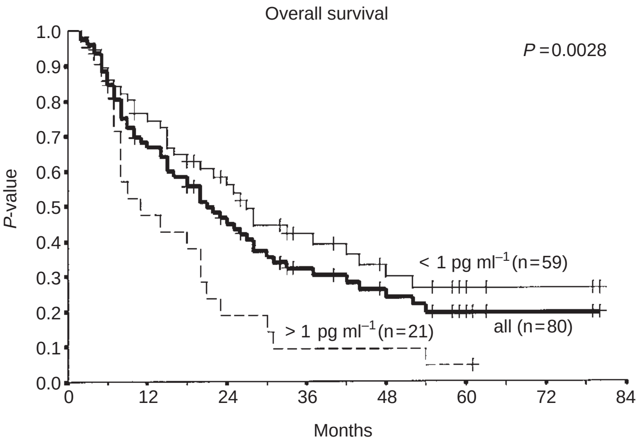

**Figure 1** Serum IL-10 and survival in 80 patients with metastatic renal cell carcinoma. Survival curves (Kaplan–Meier estimate) of patients with either low (≤ 1 pg ml⁻¹) or elevated (> 1 pg ml⁻¹) serum levels of IL-10. *P*-value was determined by log rank test. Tick marks represent patients for whom data were censored.

statistically independent: haemoglobin < 10 g dl⁻¹ ($P$ = 0.0005), ESR ≥ 50 mm h⁻¹ ($P$ = 0.049), serum LDH ≥ 240 U l⁻¹ ($P$ = 0.0233) and serum IL-10 > 1 pg ml⁻¹ ($P$ = 0.0226). The hazard ratios calculated with a model containing these prognostic factors are shown in Table 3.

### DISCUSSION

Our study demonstrated that elevated pretreatment serum IL-10 is a potentially powerful and independent predictor of overall survival in patients with metastatic renal cell carcinoma. Both by univariate and multivariate analysis, serum IL-10 ($P$ = 0.0226), serum LDH ($P$ = 0.0233), haemoglobin ($P$ = 0.0005) and ESR ($P$ = 0.049) were predominant independent predictors of survival; by demonstrating statistic independence, IL-10 adds new information to previously identified risk factors.

© Cancer Research Campaign 1999

**Table 3** Hazard ratios associated with pretreatment predictors of overall survival in a multivariate analysis of 80 consecutive patients with metastatic renal cell carcinoma, using Cox proportional hazards models

| Variable | Categories compared[a] | Hazard ratio (95% confidence interval) | *P*-value[b] |
|---|---|---|---|
| Clinical factors | | | |
| LDH (U $l^{-1}$) | < 240 vs. ≥ 240 | 0.48 (0.26–0.89) | 0.0233 |
| ESR (mm $h^{-1}$) | < 50 vs. ≥ 50 | 0.31 (0.15–0.65) | 0.049 |
| Haemoglobin (g $dl^{-1}$) | ≥ 10 vs. < 10 | 0.20 (0.09–0.45) | 0.0005 |
| Interleukin 10 (pg $ml^{-1}$) | ≤ 1 vs. > 1 | 0.50 (0.26–0.89) | 0.0226 |

[a]For each variable, the prognostic significance of the first category listed was assessed by comparing that category with the reference category (the second category listed). [b]For the comparison of the hazard ratio shown with a hazard ratio of 1.0 (as postulated by the null hypothesis).

We have shown previously that elevated serum LDH, elevated ESR and decreased haemoglobin may correlate with tumour load and with poor outcome in patients with metastatic renal cell carcinoma (Lopez-Hänninen et al, 1995).

IL-10 has been detected in different human tumours, e.g. malignant melanoma and renal cell carcinoma (Wang et al, 1995). The location of the compartment of IL-10 secretion remains to be identified in humans with malignant disease. Tumour-infiltrating lymphocytes and tumour cells of renal cell carcinoma themselves have been detected as source for IL-10 (Dummer et al, 1996; Knoefel et al, 1997). The reason for the elevated IL-10 production still remains unclear. The elevated serum IL-10 levels that we found may be correlated with a high intratumoral IL-10 production. As Buer et al (1998) recently demonstrated, IL-10 can be produced by anergic T-lymphocytes and is able to contribute to T-cell anergy. Intratumorally produced IL-10 may therefore lead to anergy of tumour-infiltrating lymphocytes and thereby support intratumoral tolerance. This may be a reason for rapid progression of some patients with malignant disease.

Other malignant diseases have been demonstrated to show a similar mechanism. Thus, elevated serum IL-10 levels have been demonstrated as a negative prognostic factor in patients with active non-Hodgkin's lymphoma (Blay et al, 1993).

In conclusion, in the present study, elevated pretreatment serum levels of IL-10 correlate with poor outcome in renal cell carcinoma. These data may contribute to a better patient selection via risk stratification for future therapeutic trials. This factor may potentially influence the decision-making of available immunotherapeutic strategies of patients with metastatic renal cell carcinoma. In addition, an improved understanding of the pretreatment immunological status of patients with metastatic renal cell carcinoma may provide a lead for future therapeutic strategies focusing on the modulation of this cytokine and/or its receptor structure. Our results will have to be prospectively confirmed in future controlled studies.

### REFERENCES

Atzpodien J, Lopez-Hänninen E, Kirchner H, Bodenstein H, Pfreundschuh M, Rebmann U, Metzner B, Illiger HJ, Niesel T and Poliwoda H (1995) Multiinstitutional home-therapy trial of recombinant human Interleukin-2 and Interferon-α2 in progressive metastatic renal cell carcinoma. *J Clin Oncol* **13**(2): 497–501

Blay J-Y, Burdin N, Rousset F, Lenoir G, Biron P, Philip T, Banchereau J and Favrot MC (1993) Serum Interleukin-10 in non Hodgkin's lymphoma: a prognostic factor. *Blood* **82**: 2169–2174

Buelens C, Verhasselt V, De Groote D, Thielemans K, Goldman M and Willems F (1997) Interleukin-10 prevents the generation of dendritic cells from human peripheral blood mononuclear cells cultured with interleukin-4 and granulocyte–macrophage-colony-stimulating factor. *Eur J Immunol* **27**: 756–762

Buer J, Lanoue A, Franzke A, Garcia C, v. Boehmer H and Sarukhan A (1998) Interleukin-10 secretion and impaired effector function of MCH class ii-restricted T cells anergized in vivo. *J Exp Med* **187**: 177–183

deWaal Malefyt R, Haanen J and Spits H (1991) Interleukin 10 and viral IL-10 strongly reduce antigen-specific human T cell proliferation by diminishing the antigen-presenting capacity of monocytes via downregulation of class II major histocompatibility complex expression. *J Exp Med* **174**: 915–924

deWaal Malefyt R, Yssel H and deVries JE (1993) Direct effect of IL-10 on subsets of human CD4+ T cell clones and resting T cells. *J Immunol* **150**: 4754–4765

Ding L, Linsley P and Huang LY (1993) IL-10 inhibits macrophage costimulatory activity by selectively inhibiting the up-regulation of B7-expression. *J Immunol* **151**: 1224–1234

Dummer W, Bastian BC, Ernst N, Schanzle C, Schwaaf A and Brocker EB (1996) Interleukin-10 production in malignant melanoma: preferential detection of Interleukin-10-secreting tumor cells in metastatic lesions. *Int J Cancer* **66**(5): 607–610

Fiorentino DF, Bond MW and Mosmann TR (1989) Two types of mouse T helper cells. IV. Th2 clones secrete a factor that inhibits cytokine production by Th1 clones. *J Exp Med* **170**: 2081–2095

Kaplan EL and Meier P (1958) Non-parametric estimation from incomplete observations. *J Am Stat Assoc* **53**: 457–481

Knoefel B, Nuske K, Steiner T, Junker K, Kosmehl H, Rebstock K, Reinhold D and Junker U (1997) Renal cell carcinomas produce IL-6, IL-10, IL-11 and TGF-beta1 in primary cultures and modulate T-lymphocyte blast transformation. *J Interferon Cytokine Res* **17**: 95–102

Kruger-Krasagakes S, Krasagakis K, Garbe C, Schmitt E, Huls C, Blankenstein T and Diamantstein T (1994) Expression of Interleukin-10 in human melanoma. *Br J Cancer* **70**: 1182–1185

Lopez-Hänninen E, Kirchner H and Atzpodien J (1995) Interleukin-2 based home therapy of metastatic renal cell carcinoma: risks and benefits in 215 consecutive single institution patients. *J Urol* **155**(1): 19–25

Matsuda M, Salazar F, Petersson M, Masucci G, Hansson J, Pisa P, Zhang QJ, Masucci MG and Kiessling R (1994) Interleukin-10 pretreatment protects target cells from tumor- and allo-specific cytotoxic T cells and downregulates HLA class I expression. *J Exp Med* **180**: 2371–2376

Nakagomi H, Pisa P, Pisa EK, Yamamoto Y, Halapi E, Backlin K, Juhlin C and Kiessling R (1995) Lack of interleukin-2 (IL-2) expression and selective expression of IL-10 mRNA in human renal cell carcinoma. *Int J Cancer* **63**: 366–371

Suzuki T, Tahara H, Narula S, Moore KW, Robbins PD and Lotze MT (1995) Viral Interleukin-10 (IL-10), the human herpes virus 4 cellular IL-10 homologue, induces local anergy to allogeneic and syngeneic tumors. *J Exp Med* **182**: 477–486

Wang Q, Redovan C, Tubbs R, Olencki T, Klein E, Kudoh S, Finke J and Bukowski RM (1995) Selective cytokine gene expression in renal cell carcinoma tumor cells and tumor-infiltrating lymphocytes. *Int J Cancer* **61**: 780–785

Yue FY, Dummer R, Geertsen R, Hofbauer G, Laine E, Manolio S and Burg G (1997) Interleukin-10 is a growth factor for human melanoma cells and downregulates HLA class-I, HLA class-II and ICAM molecules. *Int J Cancer* **71**: 630–637

 © Cancer Research Campaign 1999

## References

[PDF_00235] Atzpodien J., Lopez Hänninen E., Kirchner H., Bodenstein H., Pfreundschuh M., Rebmann U., Metzner B., Illiger H. J., Jakse G., Niesel T. (1995). Multiinstitutional home-therapy trial of recombinant human interleukin-2 and interferon alfa-2 in progressive metastatic renal cell carcinoma.. J Clin Oncol.

[PDF_00240] Blay J. Y., Burdin N., Rousset F., Lenoir G., Biron P., Philip T., Banchereau J., Favrot M. C. (1993). Serum interleukin-10 in non-Hodgkin's lymphoma: a prognostic factor.. Blood.

[PDF_00243] Buelens C., Verhasselt V., De Groote D., Thielemans K., Goldman M., Willems F. (1997). Interleukin-10 prevents the generation of dendritic cells from human peripheral blood mononuclear cells cultured with interleukin-4 and granulocyte/macrophage-colony-stimulating factor.. Eur J Immunol.

[PDF_00256] Ding L., Linsley P. S., Huang L. Y., Germain R. N., Shevach E. M. (1993). IL-10 inhibits macrophage costimulatory activity by selectively inhibiting the up-regulation of B7 expression.. J Immunol.

[PDF_00259] Dummer W., Bastian B. C., Ernst N., Schänzle C., Schwaaf A., Bröcker E. B. (1996). Interleukin-10 production in malignant melanoma: preferential detection of IL-10-secreting tumor cells in metastatic lesions.. Int J Cancer.

[PDF_00263] Fiorentino D. F., Bond M. W., Mosmann T. R. (1989). Two types of mouse T helper cell. IV. Th2 clones secrete a factor that inhibits cytokine production by Th1 clones.. J Exp Med.

[PDF_00272] Krüger-Krasagakes S., Krasagakis K., Garbe C., Schmitt E., Hüls C., Blankenstein T., Diamantstein T. (1994). Expression of interleukin 10 in human melanoma.. Br J Cancer.

[PDF_00278] Matsuda M., Salazar F., Petersson M., Masucci G., Hansson J., Pisa P., Zhang Q. J., Masucci M. G., Kiessling R. (1994). Interleukin 10 pretreatment protects target cells from tumor- and allo-specific cytotoxic T cells and downregulates HLA class I expression.. J Exp Med.

[PDF_00282] Nakagomi H., Pisa P., Pisa E. K., Yamamoto Y., Halapi E., Backlin K., Juhlin C., Kiessling R. (1995). Lack of interleukin-2 (IL-2) expression and selective expression of IL-10 mRNA in human renal cell carcinoma.. Int J Cancer.

[PDF_00285] Suzuki T., Tahara H., Narula S., Moore K. W., Robbins P. D., Lotze M. T. (1995). Viral interleukin 10 (IL-10), the human herpes virus 4 cellular IL-10 homologue, induces local anergy to allogeneic and syngeneic tumors.. J Exp Med.

[PDF_00289] Wang Q., Redovan C., Tubbs R., Olencki T., Klein E., Kudoh S., Finke J., Bukowski R. M. (1995). Selective cytokine gene expression in renal cell carcinoma tumor cells and tumor-infiltrating lymphocytes.. Int J Cancer.

[PDF_00292] Yue F. Y., Dummer R., Geertsen R., Hofbauer G., Laine E., Manolio S., Burg G. (1997). Interleukin-10 is a growth factor for human melanoma cells and down-regulates HLA class-I, HLA class-II and ICAM-1 molecules.. Int J Cancer.

[PDF_00250] de Waal Malefyt R., Haanen J., Spits H., Roncarolo M. G., te Velde A., Figdor C., Johnson K., Kastelein R., Yssel H., de Vries J. E. (1991). Interleukin 10 (IL-10) and viral IL-10 strongly reduce antigen-specific human T cell proliferation by diminishing the antigen-presenting capacity of monocytes via downregulation of class II major histocompatibility complex expression.. J Exp Med.

[PDF_00254] de Waal Malefyt R., Yssel H., de Vries J. E. (1993). Direct effects of IL-10 on subsets of human CD4+ T cell clones and resting T cells. Specific inhibition of IL-2 production and proliferation.. J Immunol.

